# “Immune senescence and dormant tumor cells: reconceptualizing breast cancer recurrence as an affliction of aging and chronic inflammation”

**DOI:** 10.1097/MS9.0000000000005032

**Published:** 2026-06-09

**Authors:** Asra Amjad, Olivia Benny, Sana Gharaee, Umair Ali, Muhammad Junaid, Muddassir Khalid

**Affiliations:** a Islamic International Medical College, Rawalpindi, Pakistan (Riphah International University); b School of Medicine, Keele University, Keele, UK; c Department of Pharmacy, University of Swabi, Swabi, Pakistan; d Department of Medicine, Lady Reading Hospital, Peshawar, Pakistan; e Department of Medicine, Nishtar Medical University, Multan, Pakistan

**Keywords:** breast cancer recurrence, immunosenescence, inflammaging, liquid biopsy, minimal residual disease, senolytics, tumor dormancy

## Abstract

One of the most significant clinical challenges in breast cancer is late recurrence, especially in estrogen receptor-positive disease. The biochemical causes of long-term dormancy are still not fully understood, and conventional surveillance frequently lacks sufficient sensitivity to reliably predict relapse. The objectives of this review are to: (1) investigate the molecular connections among immunosenescence, chronic inflammation, and tumor dormancy; (2) critically assess new therapeutic and diagnostic strategies; and (3) pinpoint methodological and translational gaps for further investigation. By combining geroscience and oncologic biology, this narrative review critically summarizes contemporary research on immunological senescence, tumor dormancy, and chronic inflammation published between 2015 and 2025. For preclinical, translational, and clinical research, databases such as PubMed and Scopus were examined, with a focus on mechanistic insights, liquid biopsy technologies, and experimental therapy approaches. Using combinations of phrases like “breast cancer recurrence,” “tumor dormancy,” “immunosenescence,” “inflammaging,” “liquid biopsy,” and “senolytics,” a focused literature search was carried out across PubMed and Scopus for research published between January 2015 and September 2025. Original English-language studies, reviews, and meta-analyses that addressed the molecular, translational, or clinical aspects of immunological aging and dormancy met the inclusion criteria. When preclinical research was pertinent to human pathology, it was included. Non-peer-reviewed commentary, conference abstracts without complete data, and research unrelated to breast cancer were the exclusion criteria. Although causality has not been established, there is evidence that immunosenescence and inflammation may create suitable habitats for dormant micrometastases. While liquid biopsy platforms for detecting minimal residual disease (MRD) provide earlier relapse signals, they have questionable clinical usefulness, analytical limitations, and confounding issues related to clonal haematopoiesis. Senolytics, epigenetic modulators, and “awaken-and-kill” immunotherapies are examples of experimental approaches that show promise but also have unidentified hazards, such as off-target toxicity and unintentional acceleration of metastases. Crucially, the majority of mechanistic insights derive from studies in young murine models, which restricts their applicability to aging human populations. Equity issues remain poorly understood, particularly in environments with limited resources. Rethinking late recurrence as a chronic inflammation and aging ailment offers a unifying theory, but it must be interpreted carefully. Translation necessitates rigorous MRD-linked trial designs, prospective validation in diverse and elderly cohorts, and a methodical assessment of both safety and efficacy. Adopting these strategies too soon could backfire if limitations and confounders are not critically evaluated.

## Introduction

**Figure 1.**
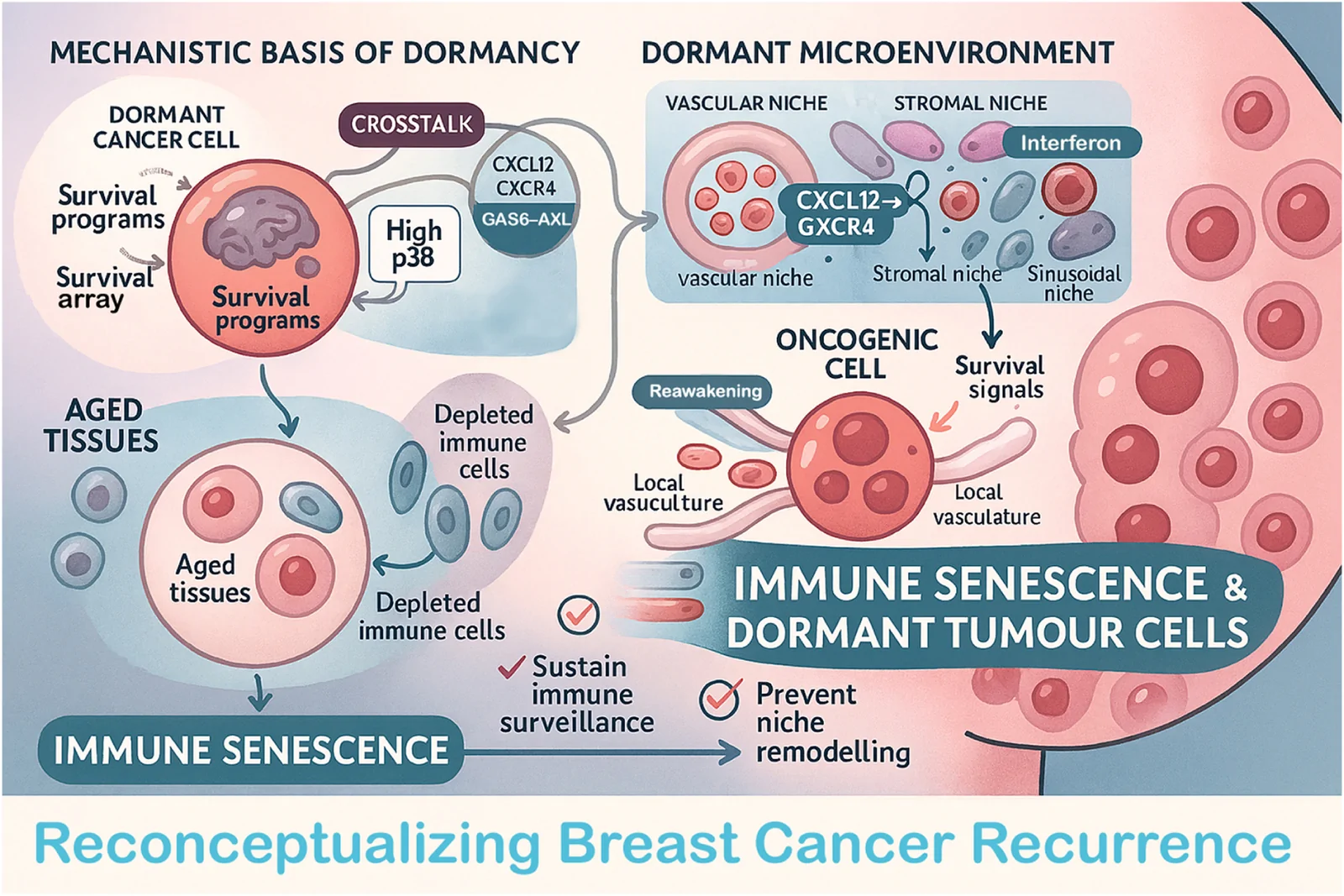
Dormant tumor cells occupy specialized niches in the bone marrow, liver, and lung, where stromal, vascular, and immune signals control dormancy entry and exit.

Despite advancements in molecular science, late recurrence remains unpredictable and unpreventable. Current frameworks continue to be tumor-centric, ignoring host aging and long-term inflammatory alterations that affect immune surveillance. By redefining recurrence as a systemic process using a geroscience lens, immune-rejuvenation and anti-inflammatory treatments may be guided beyond traditional oncologic paradigms. For clinicians, late recurrence translates into lifetime uncertainty for survivors, highlighting the need for predictive biomarkers and interventions that target host aging rather than just residual tumor biology. The survivors of breast cancer, especially those with estrogen receptor-positive (ER+) disease, are at a constant risk of recurrence even many years or decades after initial treatment^[^1^]^. The pathogenesis behind this insidious late relapse is supported by disseminated tumor cells (DTCs), which enter a prolonged dormancy and yet resist detection and conventional treatment, only to reappear under favorable conditions^[^2^]^. Notably, there are no approved therapies specifically targeting mechanisms of dormancy or minimal residual disease (MRD); hence, this clinical issue remains unresolved^[^3^]^ (Fig. 1).

Furthermore, the existing conceptual frameworks have a habit of compartmentalizing the recurrence of breast cancer into individual oncologic contexts, lacking the pivotal biological perspective of aging and chronic inflammation. The pro-tumor microenvironment is promoted by the so-called senescence-associated secretory phenotype (SASP), which is the long-term secretion of inflammatory cytokines, including IL-1β, IL-6, and IL-8. It has the potential to undermine immune defense^[^4^]^. At the same time, the immune system’s ability to monitor and contain dormant malignant cells is gradually diminished by immunosenescence, characterized by thymic involution, a decrease in naïve T-cell production, and a shift in cytokine balance^[^5^]^.

To fill these existing gaps, we hypothesize that the recurrence of breast cancer should be re-conceptualized as an aging-related inflammation phenomenon. There is growing evidence that microenvironmental permissiveness for dormant tumor reactivation is associated with inflammation and immune fatigue. Our suggested “immunosenescence-inflammation-dormancy” hypothesis is based on these convergent facts. By integrating tumor dormancy biology with geroscience and immunology in the future, it will be possible to approach the problem of dormant disease not as a reactive, but as a proactive, issue. By targeting lifelong surveillance, controlling inflammation, and promoting immune rejuvenation, it will be possible to suppress or eradicate dormant disease. This review explores the concept that this vision identifies the intersection of immune aging and chronic inflammation with tumor dormancy, and how this understanding can be translated into new paradigms of survivorship care.

TITAN Guidelines: This manuscript is compliant with the TITAN Guidelines, 2025, declaring no use of AI**^[^**6**^]^**.

### The unresolved challenge of late recurrence in breast cancer

Breast cancer, especially cases that are hormone receptor-positive, is characterized by a persistent and unpredictable threat of late recurrence – distant metastasis – which occurs 5–20 years following the initial diagnosis and effective treatment^[^7,8^]^. The results of the Early Breast Cancer Trialists’ Collaborative Group’s landmark findings show that the risk of recurrence does not decline in a linear progression long after the traditional 5-year limit, with cumulative distant relapse rates in certain groups of trialists approaching 30–40% over 20 years^[^9^]^. It is believed that DTCs lodged in niches, including the bone marrow, are the basis of this disease latency, as they enter into a dormant state, avoiding detection and traditional therapies, only to reawaken subsequently^[^10^]^. In fact, some research has revealed that about a quarter of all patients who develop breast cancer and undergo surgery succumb to systemic relapse – including late relapse – which underscores the weakness of existing surveillance procedures^[^11^]^. Accordingly, late recurrence continues to be a clinical enigma – a mystery that requires a more mechanistic understanding, a risk stratification system, and innovative monitoring paradigms to promote survivorship care.

HIGHLIGHTS

- Reorients the focus from tumor-intrinsic to host-aging biology by redefining late breast cancer recurrence as a result of immunosenescence, tumor dormancy, and chronic inflammation.
- Explains dormancy mechanisms critically, pointing out that the majority of the evidence is still associative and comes from young animal models, which limits its applicability to humans.
- Claims that although liquid biopsy and MRD detection are promising, they are currently limited by lead-time bias, clonal hematopoiesis confounders, analytical sensitivity, and unclear clinical value.
- Evaluates new treatments, such as immunological rejuvenation, senolytics, and awaken-and-kill tactics, while clearly recognizing the dangers of off-target damage and metastatic acceleration.
- Identifies equity gaps and points out that unless practical, cost-effective solutions are created, MRD and sophisticated surveillance platforms run the risk of exacerbating inequities in low-resource environments.
- Recommends that age-stratified prospective cohorts and biomarker-driven randomized studies with strict survival and safety outcomes be used before implementing MRD-triggered therapies.
- Presents the evaluation as a roadmap that is both forward-looking and cautionary, striking a balance between methodological rigor, patient safety, ethical responsibility, and innovation.

### Tumor dormancy vs. cure

The difference between cure and dormancy in breast cancer has become one of the most challenging issues in modern oncology. Traditionally, the absence of recurrence was commonly associated with a cure within 5 years of therapy; however, recent studies indicate that dormant DTCs can remain in metastatic niches for decades and potentially revitalize^[^7,8^]^. The term dormancy is not equivalent to the term eradication. Still, a dynamic balance exists between tumor cells in a dormant state, where they are not proliferating, and the immune system and the microenvironment, which restrain the growth of the tumors^[^10^]^. This so-called MRD does not fit the classic definition of remission, making it harder for clinicians to advise patients on their long-term prognosis^[^12^]^. Besides, the biological controls underpinning dormancy, e.g., signaling pathways such as p38, ERK, and TGF-β, have indicated that dormancy can be more of a regulated, reversible phenotype than an end state^[^13^]^.

### Gaps in integration: biology, clinical translation, and patient-centered frameworks

Although considerable progress has been made in investigating the biology of dormancy, its clinical application remains limited. Detailed molecular mechanisms of cellular quiescence, angiogenic control, and immune control have been elucidated; however, they are seldom incorporated into risk stratification or therapeutic interventions in patients^[^13^]^. The design of clinical trials remains primarily based on shorter recurrence endpoints, overlooking the decades-long courses that hormone receptor-positive disease typically follows, and, as such, undermining the role of late relapse^[^7^]^. In addition, technologies for detecting MRD, including circulating tumor cells (CTCs), cell-free DNA, and single-cell transcriptomics, are still largely experimental and not yet standardized for routine use^[^14^]^. Moreover, psychosocial aspects of dormancy and survivorship are understudied; patients tend to live with anxiety and insecurity due to the unseen risk of late recurrence, and health systems do not have mechanisms to monitor adverse effects after 5 years. The remedy to these gaps will involve integrative models that reconcile biological knowledge with longitudinal clinical approaches and patient-centered survivorship care, linking oncology, geoscience, and psychosocial science into a single paradigm.

Although a large portion of the mechanistic data is preclinical and associative, the conceptual synthesis presented here is convincing. It remains unclear whether inflammation and late clinical relapse are causally related; instead, other factors, such as tumor-intrinsic heterogeneity, therapy-induced selection, and genomic evolution, should be explicitly taken into account. As a result, we offer the geroscience-dormancy paradigm as a testable hypothesis that needs thorough biomarker-linked trials and prospective validation in older cohorts. Reframing recurrence as an age-related inflammatory disorder has practical implications: it promotes the use of anti-inflammatory or senotherapeutic adjuncts in survivorship care, personalized endocrine duration, and lifelong immune and inflammatory monitoring. From the standpoint of surgical oncology, redefining recurrence as an inflammatory condition associated with aging highlights the necessity of incorporating metabolic control, infection prevention, and perioperative immune modulation into survivorship planning. This methodology does not pretend to answer all ambiguities; rather, it highlights variation in biological aging and inflammatory burden among patients. Standardized biomarkers of immunosenescence and systemic inflammation are urgently needed to stratify risk in heterogeneous populations.

## Mechanistic basis of dormancy

Cancer cells in a dormant, non-dividing state arise out of dormancy. The cells maintain survival programs that enable them to remain alive without undergoing growth. The equilibrium of MAPK pathways is essential. Cell cycle arrest and dormancy are maintained by low ERK and high p38 signaling and are usually reinstated by increased ERK activity. Quiescence is further maintained by TGF-beta family niche signaling, which triggers the expression of inhibitors of cell cycle and survival genes. The cells can be locked into a stable dormant state thanks to epigenetic regulators and histone variants, while still allowing for their survival. These processes enable single DTCs to survive for an extended period^[^2,15,16^]^. Small micrometastases can remain small because they are unable to form new blood vessels. In cases of low proangiogenic signals and high levels of antiangiogenic factors, the growth of the tumor mass is inhibited. These small lesions are also characterized by hypoxia. Hypoxia alters cell metabolism and stabilizes the factors that may facilitate or inhibit angiogenesis. Tumor cells shape local vessels and remodel them to facilitate outgrowth or keep cells inactive in a niche, depending on the local environment^[^17,18^]^.

The immune system can control micro metastases. Natural killer cells and cytotoxic T cells patrol tissues, capable of imposing a dormant state through the use of an interferon signal and the cellular murder of proliferating cells. Dormant cells, however, devise several mechanisms to forestall immune clearance. The presence of low concentrations of rare cells reduces the likelihood of interacting with a specific T cell. The dormant cells are also able to manipulate antigen presentation or express checkpoint ligands to evade detection. Niche changes that cause a decrease in immune cell density or activity permit escape and outgrowth^[^16,19^]^. Aging undermines immune surveillance. Alterations in cytokine balance, loss of key niche cells, and depletion of immune cells characterize aging tissues. Such changes can reduce the barrier that holds the silent cells quiet and increase the chances of reactivation over time. It has also been found that niche aging alters local stromal cues that influence TGF-β and MAPK signaling in dormant cells^[^20–22^]^.

*In vitro* systems or young murine models, which infrequently replicate human immunosenescence or the endocrine environment of ER + disease, provide the majority of the summarized mechanistic findings. We warn against oversimplified extrapolation and suggest specific research in longitudinal human cohorts stratified by subtype and previous treatment, as well as in elderly animal models. Although these pathways are primarily derived from young or immunocompetent animal models, it is still speculative to extrapolate them to aging, hormonally changing human populations. Tumor heterogeneity, selection pressure brought on by therapy, and genetic evolution are examples of factors that probably interact with immunosenescence to influence recurrence.

## The dormant microenvironment

Specialized niches, such as endosteal or perivascular areas in bone marrow and stromal or vascular niches in the liver and lung, are home to dormant cancer cells. These habitats control whether cells stay dormant or begin multiplying. Tumor cell dormancy or reactivation is determined by molecular signals (such as CXCL12–CXCR4, GAS6–AXL, NK cell interferon, and G-CSF-induced vascular remodeling) sent by stromal, endothelial, and immune cells in these dynamic microenvironments.

While treatments seek to maintain immune surveillance, obstruct survival cues, or stop niche remodeling, crosstalk via the CXCL12-CXCR4, GAS6-AXL, and interferon pathways controls dormancy and reactivation. When dormant cells reactivate, they can rearrange the stroma and vasculature to facilitate outgrowth^[^15,19,22,23^]^. Therapeutic approaches concentrate on either initiating dormancy exit followed by eradication or sustaining dormancy through immune surveillance or suppression of survival/niche-remodeling signals. Strong preclinical promise is shown by epigenetic enforcers of dormancy and vascular signal modulation; however, clinical translation necessitates caution to prevent uncontrolled DTC activation^[^2,18,19,23^]^.

## Detection of MRD

### Circulating tumor cells and DTCs

A key process in the metastatic spread of cancer is metastasis formation. Cancer cells disseminate from the primary tumor through epithelial-to-mesenchymal transition, causing the secretion of specific enzymes that digest the extracellular matrix (ECM)^[^24^]^. Once the cancer cells have reached the bloodstream, they transform into CTCs, which are now used as biomarkers for cancer progression. CTCs form an integral part of oncological research regarding metastatic spread, specifically as a key component in liquid biopsies, which are used to monitor cancer treatment efficacy, cancer progression, and predictors of recurrence^[^25^]^. This is obtained through minimally invasive methods directly from bodily fluids, most commonly blood sources^[^26^]^. It is essential to note the limitations of liquid biopsy methods, specifically the lack of sensitivity for early cancer detection^[^27^]^, as tumor-derived biomarkers exist at low concentrations when shed into the bloodstream in early stages^[^28^]^ or are not elevated at all, such as prostate-specific antigens in advanced prostatic cancer^[^29^]^.

**Figure 2.**
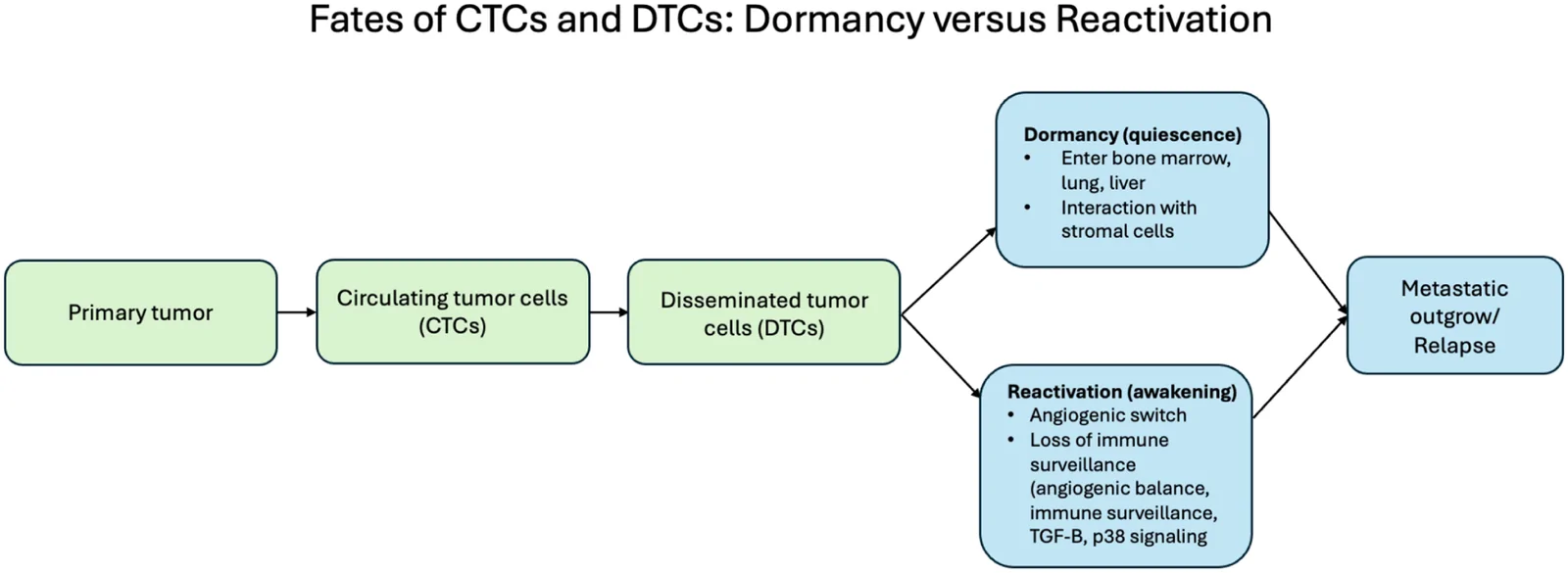
Fates of CTCs and DTCs: dormancy versus reactivation.

DTCs are slightly different in nature, as the cells travel through the bloodstream and migrate to anatomical sites, most commonly in the bone marrow (Fig. 2). It is an early stage in carcinogenesis, and the presence of early dissemination provides a strong rationale for commencing systemic treatment^[^30^]^. The detection of DTC requires more invasive procedures, such as bone marrow aspiration, which increases the risk of procedural complications, including infection. Studies have shown that patients who have undergone complete removal of the primary tumor can still present with DTC or CTC circulating, which provides a foundation for the later development of metastasis^[^31^]^.

### Circulating tumor DNA and epigenetic biomarkers

Circulating tumor DNA (ctDNA) refers to free-floating DNA molecules that are actively shed from tumor cells undergoing apoptotic or necrotic events directly into the bloodstream (Fig. 2). Clinical studies have shown that changes in ctDNA can predict responses or resistance to a given therapy (9/31) or be used in the detection of MRD^[^32^]^. An assay conducted on a cohort study involving 61 high-risk breast cancer patients found that the detection of ctDNA during monitoring had a direct association with future relapse, prompting early intervention that resulted in improved outcomes before clinical symptoms manifested^[^33^]^. There are several advantages to using ctDNA detection, including the ability to obtain data through a non-invasive approach, such as the analysis of peripheral blood^[^34^]^. However, studies have shown limitations in its use, specifically in hemato-oncological disorders, as primary tumors may shed less DNA into circulation for reasons currently unknown^[^29^]^.

In addition to changes in the genome, cancer can also be detected through epigenetic alterations, specifically in DNA methylation modifications and histone modifications via methylation/acetylation^[^35^]^. Hypomethylation, the loss of methyl groups in the genome, can affect single-copy genes and DNA sequences, causing further instability in cancer cells and contributing to carcinogenesis^[^36^]^. Deregulation of chromatin modifiers in cells can lead to the evolution of cancer cells. This occurs through epigenetic deregulation, such as mutations in chromatin-modifying enzymes and proteins that alter chromatin structure^[^37^]^.

### Single-cell and spatial transcriptomics

Personalized treatment and cancer diagnosis have been transformed by sophisticated single-cell and spatial transcriptomics. Single-cell RNA sequencing detects quiescent, stem-like cells associated with therapy resistance and recurrence^[^38^]^, as well as the significance of the tumor immune microenvironment and signaling pathways in breast cancer^[^39^]^. Studies assessing triple-negative breast cancer following pembrolizumab with or without radiation have demonstrated that spatial transcriptomics depicts interactions between latent tumor cells and the milieu, including immune and stromal cells^[^7^]^. Liquid biopsy offers potential, but it also has drawbacks such as low sensitivity, clonal hematopoiesis confounding, false positives, and unclear actionability. These issues highlight the necessity of established thresholds, ethical supervision, and informed consent prior to clinical use. Direct profiling of dormant tumor-immune interactions is now possible thanks to advanced molecular tools like single-cell and spatial transcriptomics, which link molecular dormancy signatures to immunosenescence pathways.

## Dormancy as a chronic disease paradigm

### Conceptual reframing: from cure to control

It is crucial to promote a shift in the view of breast cancer treatment and survivorship. Often, the ultimate goal for a disease is to be cured; however, in oncological settings, this endpoint is not necessarily applicable. Surgical and treatment methods (e.g., chemotherapy/radiotherapy) have been implemented in practice to “eradicate” cancerous regions; however, there is always a risk of relapse. Recurrence can occur at any time through re-emission, with strong correlations to the original staged diagnosis. For example, patients with stage T1 disease have a low risk of recurrence (~13%) with no nodal involvement^[^40^]^. With breast cancer, studies show late recurrences of cancer up to 20 years, which undermines the whole concept of “cure” for individuals. Certain types of breast cancers show very late recurrence, such as ER+ breast cancer^[^41^]^. This illustrates the concept of dormancy. Tumor dormancy is defined as the arrest of tumor growth in the primary site. It is most common in prostatic cancers. There are different forms of dormancy, such as angiogenic, cellular, and immunosurveillance-induced dormancy, which all provide distinct mechanisms of tumor dormancy^[^8^]^. Angiogenic dormancy is expressed through oncogenic Ras activation, which enhances VEGF expression and suppresses TSP, thereby stimulating tumor proliferation in a state of dormancy with insufficient blood vessel support.

### Clinical implications: lifelong monitoring and intermittent interventions

Cancer dormancy has been widely recognized as a chronic state and, therefore, can range from years to lifelong follow-up. A typical example of long-term monitoring is in patients who have had HER2-negative breast cancer, as half of the recurrences occur after 5 years post-diagnosis^[^42^]^, highlighting the importance of long-term follow-up. Several methods are used for monitoring MRD levels. Standard methods include liquid biopsies, specifically analyzing DTC, CTC, and ctDNA, which provide quantitative data on reservoirs in dormant cells^[^43^]^. Specific ctDNA longitudinal forms are instrumental in monitoring predictions of relapse in HER2-negative cases^[^44^]^ or in monitoring cancer progression in metastatic patients^[^45^]^, thereby contributing to the early detection of cancer recurrence in high-risk patients.

Evidence-based guidelines recommend surveillance after treatment of localized breast cancer through detailed history-taking, thorough breast (physical) examination, and annual mammography^[^46^]^. This is the standard protocol for follow-up; questions are posed based on the recommendation of conducting laboratory tests, such as imaging studies and circulating tumor markers, in asymptomatic patients^[^47^]^. Due to the long-term follow-up associated with recurrence, there are significant psychosocial problems attached to this, such as fear of recurrence. Such worries can contribute to the overall recurrence, with psychological stress (including forms of anxiety and depression) moderately linked to cancer recurrence; however, data are limited to draw a strong correlation between the two^[^48^]^. Therefore, it is essential to provide social support to survivors to ensure good emotional well-being and promote the use of healthy coping strategies to address negative associations with feelings of recurrence^[^41^]^.

## Triggers of dormancy escape

**Figure 3.**
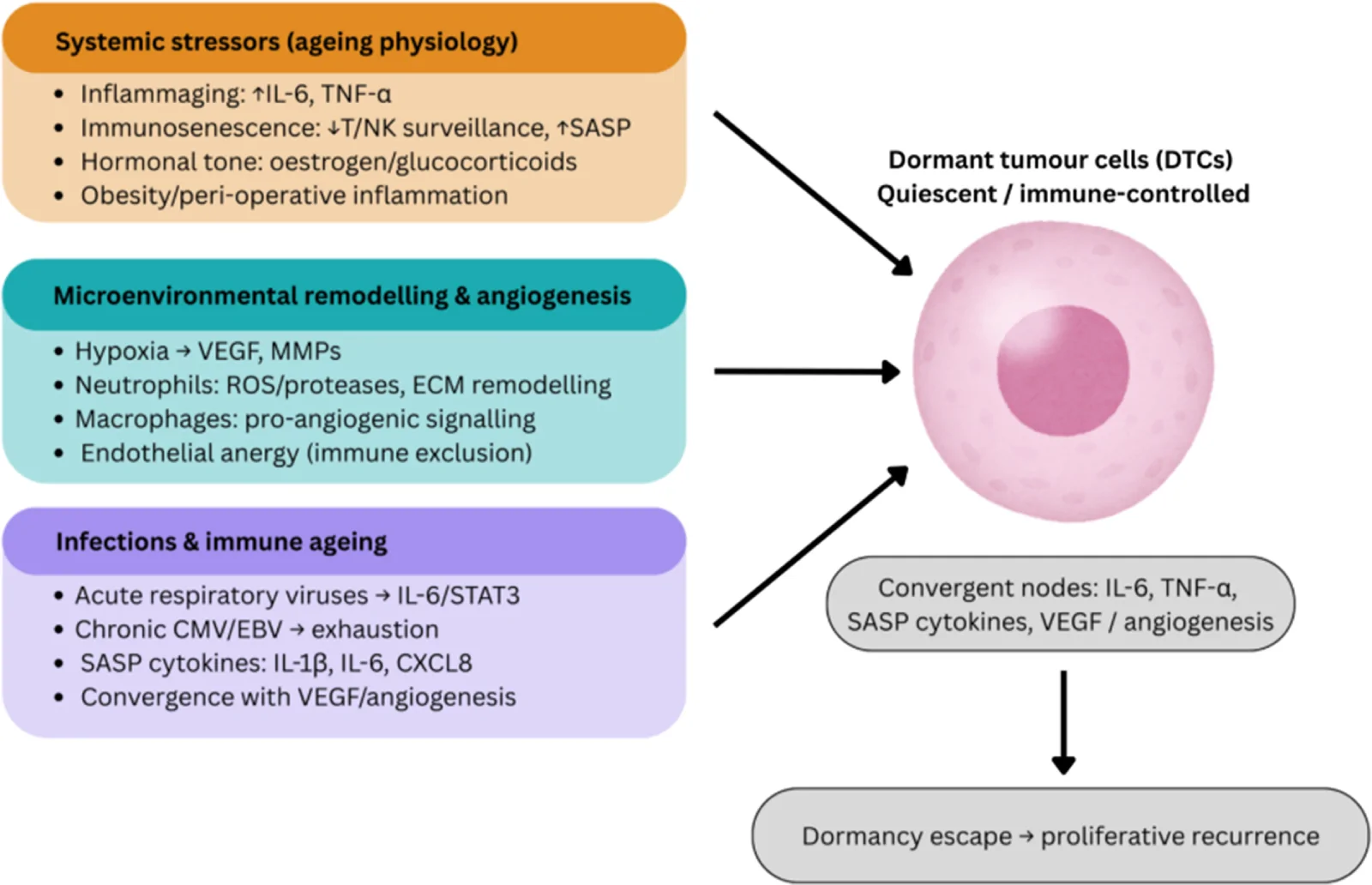
Extrinsic triggers of dormancy escape in breast cancer.

One of the key factors in breast cancer recurrence is the awakening of latent tumor cells, which turns slow micrometastases into clinically noticeable illness. Although internal programs create and preserve quiescence, escape is typically determined by the extrinsic environment, which includes immunosenescence, systemic stresses, and microenvironmental remodeling. These factors contribute to the explanation of why older individuals are disproportionately affected by late relapse and why recurrence risk remains decades after primary treatment. Crucially, they rarely work alone; instead, they combine to create an environment that is conducive to tumor formation. This interaction can be thought of as three converging domains that work together to break dormancy and encourage recurrence: infection-driven immune aging, systemic inflammatory and hormonal stresses, and local microenvironmental cues (Fig. 3).

DTCs are maintained in a quiescent, immune-controlled state until external cues destabilize this equilibrium. Microenvironmental remodeling, systemic inflammatory and hormonal stressors, and infection-driven immune aging converge through shared mediators such as IL-6, TNF-α, VEGF, and SASP cytokines to promote dormancy escape and recurrence.

### Microenvironmental remodeling and angiogenesis

DTCs, or dispersed tumor cells, live in a delicate balance with their habitats. Insufficient vascularization limits their growth during angiogenic dormancy; however, this equilibrium is disrupted when they remodel into a pro-angiogenic phenotype. Together, matrix metalloproteinases, stromal-derived factors, and hypoxia-driven VEGF release break down inhibitory barriers and promote outgrowth^[^13,49^]^. In this change, immune cells embedded in the microenvironment play a crucial role. Through the use of proteases and reactive oxygen species, neutrophils modify ECMs to create environments that facilitate invasion^[^49^]^. As gatekeepers of metastatic awakening, tumor-associated macrophages maintain vascularization and facilitate immune evasion^[^50^]^. In addition to immune-stromal communication, tumor endothelial cells themselves may develop anergic responses, which exclude effector lymphocytes and promote vascularization^[^51,52^]^. Collectively, our results demonstrate that angiogenic dormancy involves intricate interactions among stromal, vascular, and immunological factors, rather than being solely a function of vessel density. This viewpoint is confirmed by reviews, such as those by Huinen *et al*, which emphasize how vascular remodeling and endothelial immunosuppression work together to break dormancy^[^52^]^. Although local stimuli may trigger escape, systemic inflammatory forces frequently serve as catalysts for this process.

### Systemic stressors: hormones and inflammation

Systemic signals provide the physiological context against which local changes occur. Obesity and aging are associated with chronic inflammation, which causes the upregulation of IL-6, TNF-α, and other cytokines. This reprograms the immunological and stromal compartments to favor proliferation^[^53,54^]^. The term “inflammaging” refers to the way that low-grade, chronic immune activation produces a milieu rich in cytokines, which impairs tumor management and facilitates reactivation^[^55^]^. These effects are amplified by immunosenescence. The increase of senescent cells producing SASP factors, decreased T-cell repertoire variety, and compromised NK-cell cytotoxicity all work together to impair immune surveillance and provide pro-tumorigenic signals^[^56,57^]^. These systemic alterations establish a permissive baseline, allowing dormant cells to revive and proliferate. Although little researched, hormonal effects may interact with inflammatory pathways. Variations in estrogen and glucocorticoids can alter immunological tone and vascular permeability; glucocorticoid-induced immunosuppression is significant during perioperative periods. Additionally, observational studies indicate that hormonal changes and endocrine therapy may interact with latent cell types to affect recurrence risk^[^53^]^. Acute inflammatory insults, such as infection, can serve as direct triggers for reactivation when layered on top of these long-term systemic effects.

### Underrecognized drivers: infections, cytokines, and immune aging

Sudden inflammatory shocks from acute infections can trigger latent illness. A seminal work published in Nature demonstrated that respiratory viral infections, such as influenza and SARS-CoV-2, reawaken dormant breast cancer cells in the lungs by disrupting local immune regulation and IL-6–STAT3 signaling^[^58^]^. This provides strong mechanistic evidence that clinically prevalent viruses can serve as external relapse triggers. Long-term viral exposures further weaken immune surveillance. By downregulating MHC-I, expressing PD-L1, secreting TGF-β, and attracting regulatory T cells, breast cancer cells avoid immunological detection. These changes weaken cytotoxic surveillance and allow latent cell persistence, which is exacerbated by immunological aging. A cytokine milieu dominated by SASP and rich in IL-1β, IL-6, CXCL8, and VEGF is produced by persistent infections such as CMV, which hasten T-cell fatigue and immunosenescence^[^59,60^]^. To break the latent state, these elements combine with angiogenic pathways^[^56,61^]^. These observations are consolidated in reviews of the immunosenescent tumor microenvironment, which highlight how aging and infections interact to produce an inflammatory, pro-angiogenic cytokine landscape^[^59^]^. Accordingly, infections should be viewed as biological occurrences that have the power to trigger the reappearance of dormant diseases rather than as incidental comorbidities. A combination of infection-driven immunosenescence, systemic stresses, and microenvironmental remodeling breaks down dormancy. Instead of being distinct, these pathways converge at common nodes such as angiogenic signaling, TNF-α, IL-6, and SASP cytokines. It is important to use caution when interpreting the apparent causal relationship between immunosenescence, chronic inflammation, and recurrence. Although these processes are linked, they are not always sequential; host comorbidities, therapy-induced clonal selection, and genetic heterogeneity may still cause confusion. Dormancy escape is positioned as the cumulative result of local niche disturbance exacerbated by systemic inflammation and immunological decline, as shown in Figure 3, which synthesizes these triggers into a cohesive framework. This comprehensive viewpoint emphasizes the clinical fact that host aging, environmental exposures, and tumor biology influence recurrence risk. This kind of framing prepares the reader for Therapeutic strategies, which discuss therapeutic approaches to combat these triggers.

## Therapeutic strategies

Therapeutic approaches that either maintain dormancy, eradicate dormant cells, or stop their reactivation have been spurred by the realization that dormant DTCs are the primary cause of late breast cancer recurrence. These strategies include both innovative drugs undergoing preclinical or early clinical investigation and traditional therapies. The current treatment landscape is summarized in Table 1, which is arranged according to strategy type, mechanisms, representative agents, and developmental stage.

**Table 1:** Therapeutic strategies targeting breast cancer dormancy.

Strategy type	Example agents/approaches	Mechanism of action	Stage of development	Key references
Maintenance	CDK4/6 inhibitors and mTOR blockade	Induce/maintain quiescence and suppress signaling	Approved/ongoing trials	^[^1,2^]^
Elimination (“Awaken-and-Kill”)	Wnt agonists + cytotoxics	Force re-entry into the cycle and sensitize to apoptosis	Preclinical/early translational	^[^3,4^]^
Established interventions	Bisphosphonates, CDK4/6i, and checkpoint inhibitors	Alter niche, suppress proliferation, and boost immune surveillance	Phase III/clinical use	^[^5,7,8^]^
Emerging frontiers	Senolytics, lock-in, metabolic modulators, and immune rejuvenation	Eliminate SASP, arrest cells, target metabolism, and restore immune function	Preclinical/early clinical	^[^9–15^]^

### Maintenance approaches: cell-cycle inhibitors, mTOR blockade, and immune surveillance

The goal of maintenance strategies is to keep tumors dormant and stop them from reactivating. One intriguing tactic is to block cell-cycle progression: in preclinical models, CDK4/6 inhibitors preserve long-term quiescence and reduce the proliferation of latent DTCs^[^62^]^. These medications, which are already authorized for advanced breast cancer, are being investigated for their ability to lower the risk of late recurrence in the adjuvant context. Another way to extend dormancy is to target the PI3K/AKT/mTOR axis. Signaling pathways that promote DTC survival and expansion are disrupted by mTOR inhibition. Everolimus was first used as a targeted treatment for hormone receptor-positive breast cancer in seminal clinical trials like BOLERO-2. Since then, research has improved its function and integration with endocrine therapy^[^63^]^. Sustaining immune surveillance, especially in preventing dormant cells from evading detection, may also be essential for maintenance. The requirement for techniques that maintain quiescence while limiting immune escape is reflected in the evaluation of low-dose checkpoint modification and cytokine support as supplementary approaches to boost immune vigilance without generating full activation^[^64^]^. All of these results highlight the translational potential of immune surveillance, pathway blocking, and cell-cycle inhibition in preserving a suppressed state.

### Elimination approaches: awaken-and-kill strategies

Elimination tactics, as opposed to maintenance strategies, aim to eliminate dormant DTCs by causing them to re-enter the cell cycle, which makes them more susceptible to treatment. Wnt pathway agonists have been shown in preclinical research to have the ability to rouse dormant breast cancer cells, making them vulnerable to substances that induce apoptosis^[^65^]^. The problem of therapeutic resistance in quiescent cells is immediately addressed by this “awaken-and-kill” paradigm, which makes it philosophically persuasive. Frameworks for incorporating elimination techniques into clinical trial design have been proposed in recent studies, with a focus on sequential cytotoxic therapy following controlled proliferation induction^[^66^]^. However, striking a balance between safety and effectiveness is crucial, as insufficient subsequent eradication could potentially encourage the establishment of metastases if reawakening latent cells occurs. To convert the awaken-and-kill strategy into a clinically safe and successful approach, trial designs must incorporate biomarkers of dormancy, meticulous therapy sequencing, and thorough monitoring.

### Established interventions: bisphosphonates, CDK inhibitors, and immune checkpoint therapies

Dormancy biology is indirectly modulated by several therapies that are currently in clinical use. In postmenopausal women with hormone receptor-positive breast cancer, adjuvant bisphosphonate therapy has been demonstrated to decrease late recurrence, demonstrating the ability of bone-targeted medicines to modify metastatic habitats^[^67^]^. In a similar vein, CDK4/6 inhibitors are being investigated as adjuvant therapies to lower residual proliferative activity and prolong disease-free longevity, beyond their maintenance potential^[^68^]^. Immune checkpoint blockade has been shown to improve survival in high-risk early breast cancer. It may also enhance immune monitoring of dormant cells and limit escape, especially when combined with PD-1/PD-L1 inhibitors, such as pembrolizumab^[^69^]^. Together, these approaches illustrate how existing therapies can be reframed through the lens of dormancy, expanding their relevance from tumor suppression to recurrence prevention.

### Emerging frontiers: senolytics, metabolic modulators, and immune rejuvenation

Innovative biology at the nexus of dormancy, senescence, metabolism, and immunological aging is the focus of emerging therapeutic frontiers. Particularly intriguing are senolytic treatments, which eradicate senescent cells and the cytokine milieu they produce, triggered by the SASP. Senolytics can prevent dormant tumor cells (DTCs) from reactivating due to senescence, according to reviews^[^70,71^]^. The viability of this strategy in triple-negative breast cancer models is demonstrated by preclinical research that combines PARP inhibition and radiation with senolytic drugs like venetoclax^[^72^]^. An alternative “lock-in” technique offers a conceptual counterbalance to elimination approaches by attempting to permanently halt cancer cells in their dormant state rather than reawakening them^[^73^]^. Because inactive cells depend on specific adaptations, such as fatty acid oxidation, which can be therapeutically targeted, metabolic modulators also show promise^[^74^]^. Lastly, immunological rejuvenation techniques aim to re-establish the surveillance capabilities that immunosenescence has weakened, placing dormancy at the intersection of geroscience and cancer^[^75^]^. By emphasizing dormancy as a modifiable disease state, these new therapies expand the therapeutic toolkit beyond conventional approaches. Senolytics and immune rejuvenation therapies are promising paradigms, but their off-target effects, such as potential metastasis acceleration or immune dysregulation, call for strict safety monitoring and limited use within controlled clinical trials. Crucially, dormancy management can shift from passive observation to proactive, individualized intervention by implementing maintenance, eradication, established, and frontier approaches in combination or sequentially. These strategies are complementary rather than mutually exclusive (Table 1).

The table summarizes approaches designed to maintain tumor cell quiescence, eliminate dormant DTCs, or prevent their reactivation. Strategies are organized by category, with representative agents, mechanisms of action, stage of clinical development, and key references.

Strategies like “awaken-and-kill” and senolytics have potential hazards that are not insignificant (e.g., partial reactivation fuelling outgrowth or off-target toxicity). In our opinion, any first-in-human trials should be limited to clearly defined high-risk groups and should include biomarker-based selection, safety stopping rules, and emergency rescue protocols. To show net benefit, randomized, biomarker-stratified designs with MRD endpoints (and clinically meaningful survival endpoints) are needed.

## Psychosocial, ethical, and equity dimensions

### Patient anxiety and uncertainty around MRD

The presence of MRD is an invisible disease burden in patients who have survived beyond the initial breast cancer treatment, and existing clinical instruments cannot accurately rule out the presence of any dormant DTCs. This diagnostic uncertainty causes a great deal of psychological distress, mainly because patients know a relapse can occur many years after they have supposedly gone into remission^[^76^]^. Research continues to indicate that fear of recurrence is among the most common and chronic psychosocial issues among breast cancer survivors, and MRD uncertainty amplifies this fear^[^77^]^. The absence of standardized MRD testing or validated biomarkers further complicates the anxiety of patients who are thrust into survivorship without much knowledge of their actual risk^[^3,78^]^. In addition, younger survivors and individuals with hormone receptor-positive disease – who have a long risk of late recurrence – report particularly elevated rates of persistent vigilance and cancer-related worry^[^78^]^. The solution to this dilemma must be sought through the incorporation of psychosocial support, open communication about the symptoms of dormancy biology, and survivorship programs that include the normalization of long-term surveillance. In the absence of such structures, MRD uncertainty poses a significant risk not only to emotional health but also to compliance with surveillance and treatment, and ultimately, survivorship^[^79^]^.

### Ethical considerations in MRD disclosure

The recent emergence of sensitive technologies to identify MRD, including ctDNA assays and single-cell methods, has raised urgent ethical concerns regarding how to disclose and manage patients. Although early MRD diagnosis provides the potential to act before the disease progresses, the lack of adequate treatment to address MRD makes disclosure ethically challenging: telling patients they have MRD but do not have a choice to act is likely to result in distress and poor quality of life^[^14^]^. Ethical principles should be able to reconcile the principles of beneficence, which implies the provision of information that may assist in closer surveillance or the patient’s participation in a clinical trial, and non-maleficence, which warns against needless psychological harm^[^80^]^. Additionally, doubts about the accuracy of MRD assays (such as false positives and false negatives) make informed consent more difficult and complicate shared decision-making^[^81^]^. The autonomy of patients means that they require clear communication on the potential value of MRD results, as well as their limitations. However, research shows that there is a significant divergence in the way oncologists undertake such communications^[^82^]^. In addition to personal experiences, the implementation of MRD disclosure into clinical practice may lead to broader issues of justice and fairness, where more expensive diagnostics can be inaccessible in low-resource facilities, thereby exerting increased differentiation on survivorship services^[^83^]^.

### Addressing global inequities in dormancy research

Research on tumor dormancy and MRD has been growing rapidly in high-income countries. However, there are still marked global disparities in research capabilities and access to emerging technologies. High-tech methods, such as ctDNA, single-cell sequencing, and spatial transcriptomics, are mostly confined to resource-intensive environments and are therefore inaccessible to low- and middle-income countries (LMICs) for participation^[^84^]^. Such an imbalance creates a two-level terrain, where innovative understanding of dormancy biology and observation are developed in a single setting. Yet, it cannot accurately reflect the genetic, ecological, and health-system realities of worldwide patient groups. In LMICs, survivors are also disadvantaged by a lack of surveillance over more extended periods, endocrine treatment, and psychosocial support structures, which further strengthen inequity in recurrence consequences^[^85^]^. To develop equitable dormancy research, it is necessary to invest specifically in global partnerships, capacity-building, and technology transfer, as well as to have policies that promote inclusivity in clinical trials. In the absence of such measures, the gains in the science of dormancy would be used to strengthen rather than exacerbate the existing inequalities in cancer survivorship^[^86^]^.

## Future directions

Instead of being driven by intellectual fervor, future studies on breast cancer dormancy and recurrence must be grounded in methodological rigor. Before their clinical use, standardization and validation of MRD assays are the top priorities. If test sensitivity, thresholds, and confirmation procedures are not standardized to rule out clonal hematopoiesis, MRD will produce more noise than useful information. There is a pressing need for prospective, age-stratified cohort studies, as a large portion of the existing information comes from young animal models that cannot accurately mimic immunosenescence or the endocrine milieu of older adults – precisely the individuals who are at risk of late relapse. Additionally, translational success necessitates a move to older, immunologically relevant preclinical systems that fully disclose sex, age, and previous therapy exposure. A methodical clinical approach ought to be used. Translational success depends on integrating elderly, postmenopausal, and ethnically diverse cohorts into MRD-linked clinical studies, given the mismatch between preclinical and human aging models. Until randomized, biomarker-stratified studies show enhanced survival and safety, MRD-guided therapies should not be used outside of trials. Before moving on to high-risk, irreversible techniques like senolytics or “awaken-and-kill” medicines, early-phase trials should evaluate lower-risk, reversible procedures, including optimized endocrine therapy, metabolic regulation, and anti-inflammatory tactics. To identify harm, including the potential risk of accelerated metastases, trial designs must include rigorous safety stopping guidelines, early rescue procedures, and objective clinical outcomes. Integrating cost-effectiveness analyses, patient-reported outcomes, and assessments of psychological distress from false positives is equally important. Low-resource environments should be addressed via parallel pragmatic paths to prevent global disparities from growing as a result of advancements in dormancy biology and MRD detection. Lastly, direct disclosure policies, data sharing, and the moral application of MRD in survivorship care, registries, standardized endpoints, and early interaction with regulators and ethicists will be essential.

## Conclusion

The idea that tumor dormancy, immunosenescence, and persistent inflammation interact to cause late breast cancer recurrence is a strong one. However, the majority of the supporting data remains associative, and the translational claims are based on models that are not well-suited to human aging. Although liquid biopsy technologies have potential as early indicators of relapse, caution is necessary due to their questionable actionability, lead-time bias, and analytical constraints. Similarly, the very real risk of injury from early use of senolytics and immunological reawakening must temper therapeutic optimism centered on these therapies. The path forward requires disciplined progression: harmonizing assays, validating prognostic and predictive value in diverse, aged cohorts, and testing interventions only in rigorously designed, biomarker-driven trials with survival and safety endpoints. Anything less risks conflating hypothesis with evidence and advocacy with science. If the field succeeds in marrying ambition with caution, it could not only redefine how recurrence is predicted and prevented but also provide a model for aging-informed oncology. If it fails, however, it will add to the long list of premature innovations that promised more than they could deliver and left patients worse off. The distinction between progress and overreach will depend on whether critical appraisal remains at the center of the research agenda.

## Data Availability

No new data were generated or analyzed in this study. All data discussed are from published sources cited within the manuscript.

## Peer and provenance statement

Not commissioned; externally peer-reviewed.
